# Case report of oxalate nephropathy in a patient with pancreatic metastases from renal carcinoma

**DOI:** 10.1186/s12885-019-6215-y

**Published:** 2019-10-17

**Authors:** Karin Purshouse, Sarah Chamberlain, Maria Soares, Mark Tuthill, Andrew Protheroe, David R. Mole

**Affiliations:** 1 Department of Oncology, Churchill Hospital, Oxford University Hospitals NHS Foundation Trust, Oxford, OX3 7LE UK; 2 Department of Cellular Pathology, John Radcliffe Hospital, Oxford University Hospitals NHS Foundation Trust, Oxford, OX3 9DU UK; 3 Oxford Kidney Unit, John Radcliffe Hospital, Oxford University Hospitals NHS Foundation Trust, Oxford, OX3 9DU UK; 4 NDM Research Building, University of Oxford, Old Road Campus, Headington, Oxford, OX3 7FZ UK

**Keywords:** Renal cancer, Pancreatic metastases, Renal impairment, Oxalate nephropathy

## Abstract

**Background:**

Patients with metastatic renal carcinoma frequently have pre-existing renal impairment and not infrequently develop worsening renal function as a complication of their treatment. The presence of pancreatic metastases in patients with metastatic renal carcinoma, often confers a more favourable prognosis and as a consequence this patient group may be exposed to such treatments for more prolonged periods of time. However, the development of renal failure may also be a consequence of the cancer itself rather than its treatment.

**Case presentation:**

We present an 84-year-old patient receiving the tyrosine kinase inhibitor (TKI) pazopanib for metastatic renal carcinoma who developed oxalate nephropathy as a consequence of pancreatic exocrine insufficiency resulting from pancreatic metastases.

**Conclusions:**

This case demonstrates the importance of investigating unexpected toxicities and highlights the potential consequences of pancreatic insufficiency and its sequelae in patients with pancreatic metastases.

## Background

Over 400,000 people worldwide are newly diagnosed with renal cancer each year [1] and while 20–30% present with metastatic disease, up to 50% of patients will develop metastases following nephrectomy [2]. Age is a key risk factor, with incidence rates in the UK highest in those between 85 and 89 years old, and these patients are more likely to have other comorbidities (CRUK, 2019). Consequently, it is more likely for newly diagnosed patients to present with other medical problems that increase the complexity of their care.

We present an unusual cause of renal failure in a patient undergoing systemic treatment for metastatic renal carcinoma with the tyrosine kinase inhibitor (TKI) pazopanib. Over the course of 30 months, he demonstrated a good response to treatment but developed progressive renal failure, eventually commencing haemodialysis. The unexpected cause of his renal failure demonstrates the importance of critically evaluating seemingly benign symptoms on TKIs and pursuing the true pathology.

## Case report

An 84-year-old man who had previously undergone a right radical nephrectomy for renal cell carcinoma presented to his general practitioner 15 years later with iron deficiency anaemia (haemoglobin 95 d/dL, mean cell volume 79.2 fl, ferritin 16.5 μg/l, transferrin saturation 6%). His co-morbidities included type 2 diabetes mellitus, asthma, ischaemic heart disease (coronary artery bypass graft 13 years previously) and a hip replacement. His medication included ramipril, bisoprolol, simvastatin, metformin, aspirin, vitamin B12, ferrous sulphate and a fentanyl patch.

An oesophago-gastroduodenoscopy (OGD) revealed an ulcerating duodenal mass, and the biopsy confirmed metastatic clear cell renal carcinoma. A CT scan demonstrated the large 110 mm duodenal mass was centred in the head of the pancreas and was causing gastric outlet obstruction with mild pancreatic duct dilatation (Fig. 2). His amylase was 23 IU/L. In addition, there were multiple, bilateral pulmonary metastases, and a 25 mm left renal nodule in keeping with a second renal tumour.

At his initial oncology assessment his ECOG performance status was 1, he was living independently and was managing all activities of daily living without assistance. He therefore commenced palliative systemic therapy with pazopanib at a dose of 800 mg once daily as first-line treatment for his metastatic renal cell carcinoma. A re-staging CT scan after 3 months of treatment indicated disease response. However, he developed reduced appetite, grade 2 diarrhoea and grade 3 fatigue and his ECOG performance status deteriorated to 3. Therefore, after a short treatment break, his pazopanib was reduced to 400 mg once daily. A further CT scan after 6 months of treatment demonstrated ongoing disease response, and at that time his only persisting toxicity remained grade 1–2 diarrhoea, which was managed with loperamide.

**Fig. 1:**
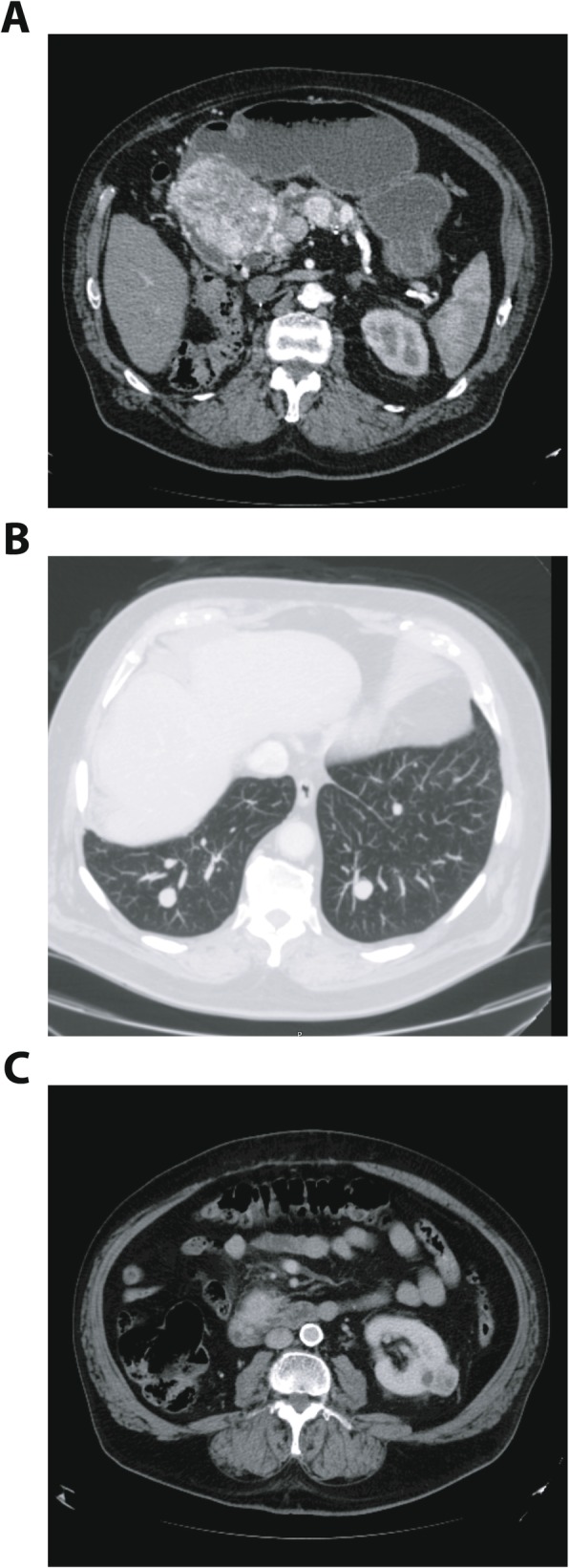
CT scan at re-presentation (**a**) 11 cm enhancing mass in the head of the pancreas, compressing the duodenum and leading to dilatation of the pancreatic duct with two smaller lesions in the body of the pancreas. **b** Multiple bilateral pulmonary metastases. **c** Exophytic lesion in the left kidney

**Fig. 2:**
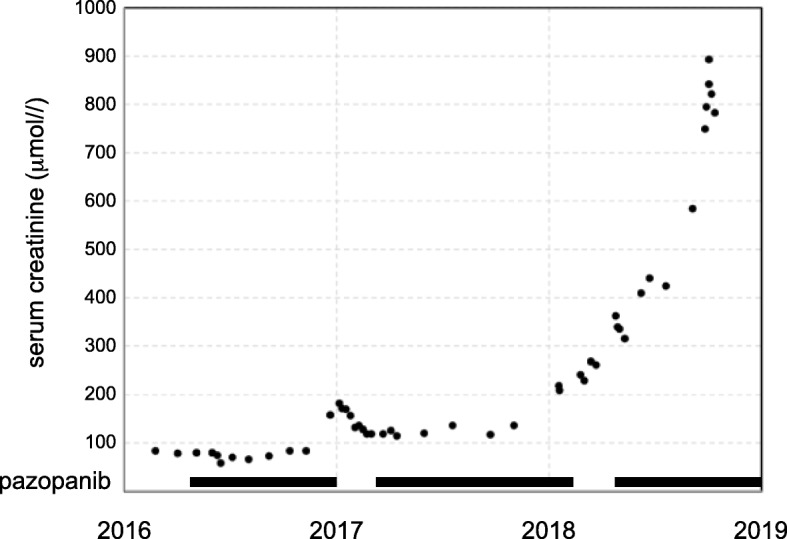
Graph of serum creatinine against time. Black bars represent the periods during which the patient took pazopanib

However, after 9 months of treatment, he developed a severe bout of diarrhoea, accompanied by dehydration and severe postural hypotension. His renal function deteriorated (Fig. 1 – Point C) and his creatinine rose from a baseline of 84 μmol/L to 158 μmol/L (Fig. 2) and his estimated glomerular filtration rate (eGFR) fell from 80 ml/min/1.73m^2^ to 37 ml/min/1.73m^2^. An ultrasound scan of his single remaining kidney revealed no evidence of obstruction. Urinalysis was negative for blood and a urine-to-creatinine ratio of 16 mg/mmol demonstrated negligible proteinuria. Serum electrophoresis and immunoglobulins, auto-antibody titres and complement levels were all unremarkable.

A working diagnosis of acute kidney injury (AKI) from acute tubular necrosis (ATN) due to hypovolaemia and hypertension as a result of his diarrhoea was made. His pazopanib and antihypertensive agents were temporarily withheld and he was rehydrated. His renal function improved and his creatinine fell to 119 μmol/L and his eGFR rose to 53 ml/min/1.73m^2^, although failed to return to his previous baseline. Given the ongoing response of his metastatic RCC to pazopanib, the drug was re-introduced. Over the ensuing year his eGFR remained stable on this medication with continued oncological response.

Following this period of stable renal function, his creatinine subsequently began to progressively rise again. This time, there was no identifiable disruption of fluid balance, haemodynamic disturbance or exacerbation of his gastrointestinal symptoms. Other than pazopanib, he was not taking any potentially nephrotoxic agents. There was only moderate proteinuria (uPCR was 56.3 mg/mmol) and further CT imaging again excluded renal obstruction.

**Fig. 3:**
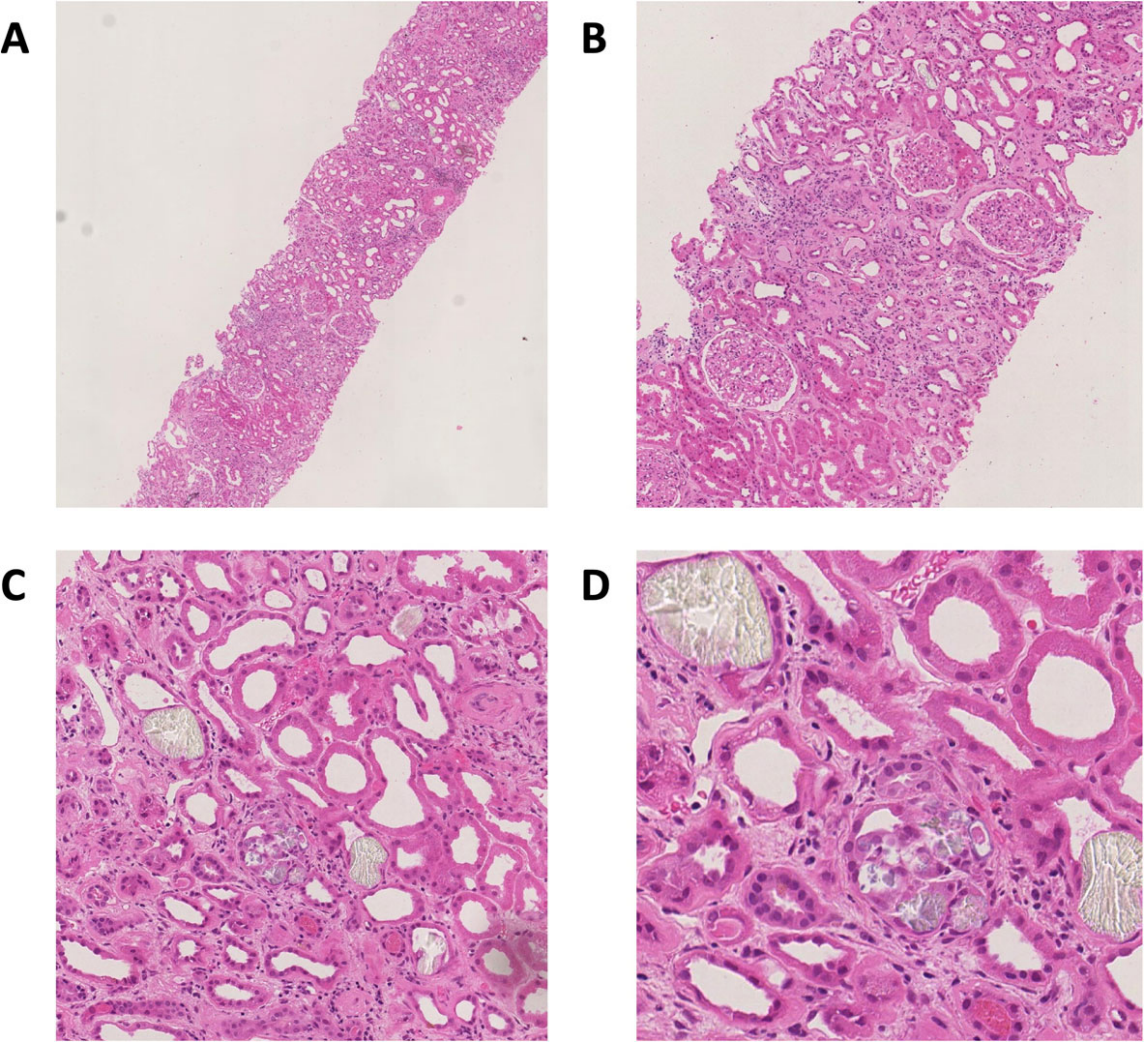
Renal biopsy stained with haematoxylin and eosin. **a** 40x magnification - Renal cortex showing moderate to severe chronic damage (50–60% interstitial fibrosis and tubular atrophy). **b** 100x magnification showing detail of cortex highlighting banded distribution of interstitial fibrosis and tubular atrophy and entrapment of normal glomeruli. **c** 200x and (**d**) 400x magnification showing proximal tubule epithelium, which is flattened with complete and partial loss of the brush borders, in keeping with acute tubular injury. A few tubular lumens contain colourless, polygonal, refractile crystals, in keeping with oxalate crystals

A renal biopsy including immunofluorescence and electron microscopy demonstrated mild acute tubular necrosis (ATN), severe chronic tubulointerstitial damage, with 60% interstitial fibrosis and tubular atrophy (IFTA) and a disproportionately large number of interstitial oxalate crystals (Fig. 3). There was no evidence of an active glomerular lesion (other than mild diabetic changes) or thrombotic microangiopathy to suggest a tyrosine kinase nephropathy. Subsequent 24-h urinary oxalate analysis demonstrated raised urinary oxalate excretion - urine oxalate 658 μmol/L, 645 μl/24 h and a urine oxalate:creatinine ratio of 153umol/mmol. His faecal elastase-1 level was reduced at 61 μg/g consistent with pancreatic exocrine insufficiency. Taken together, these findings were suggestive of secondary oxalate nephropathy from pancreatic exocrine insufficiency due to his pancreatic metastatic lesions as the primary cause of his renal impairment.

Pancreatic supplementation with oral creon was commenced, which resulted in significant improvement in his gastrointestinal symptoms. He also took oral calcium acetate to act partially as a “phosphate binder”, but also to help chelate oxalate in the bowel and reduce its absorption. His renal function continued to deteriorate and he subsequently commenced maintenance haemodialysis for end-stage renal failure (Fig. 1). Thirty months after starting treatment for metastatic RCC, he remains on first-line pazopanib with repeat imaging demonstrating only very minor disease progression in the pancreas and left kidney and stable lung metastases.

## Discussion and conclusions

Renal impairment is common in patients taking systemic therapy for metastatic RCC [3–5]. Firstly, patients may have pre-existing renal impairment. Indeed, RCC is more common in patients with chronic kidney disease than in the general population as a whole. Secondly, many patients will have had prior resection of their primary tumours (often as an attempted curative procedure in patients who subsequently develop metachronous metastases) and will have lost varying degrees of renal mass as a consequence of this surgery. Furthermore, patients may also develop worsening renal impairment during the course of their treatment. In these patients, one important distinction is to determine whether any progressive renal impairment is caused by the treatment (e.g. tyrosine kinase nephropathy, immune-mediated disease with PDL-1 directed therapies), by the cancer itself (e.g. paraneoplastic membranous glomerulonephritis) or by unrelated factors, since this may have implications, not only for managing the renal impairment, but also for the continued management of their cancer.

Here we present an unusual cause of renal impairment, due to oxalate nephropathy, in a patient with metastatic RCC involving the pancreas. This is important to recognise, since patients with pancreatic metastases often have a more indolent progression of their cancer than other patients with metastatic RCC and once established, the progressive renal impairment may be difficult to reverse or arrest. Furthermore, any response to treatment may be insufficient to relieve pancreatic duct obstruction and even when this does occur, residual pancreatic fibrosis and/or atrophy may lead to permanent exocrine insufficiency [6]. It also illustrates the importance of taking a good history from the patient since in retrospect the diarrhoea that was attributed to the common side-effect seen with tyrosine kinase inhibitors had several features of steatorrhoea.

### Oxalate nephropathy

Oxalate is an end-product of amino-acid metabolism that is produced via the glyoxylate and ascorbic acid pathways [7]. It is also absorbed from the stomach, small bowel and colon [8]. In the steady state, this is matched by (renal) excretion and therefore hyperoxaluria can be caused by either increased production or increased absorption. Increased production may result from inherited enzyme deficiencies - primary hyperoxaluria [7], increased precursor levels - e.g. from ingestion of ethylene glycol [9], vitamin B6 deficiency states [10] such as chronic alcohol abuse or from excess Vitamin C ingestion [11].

Increased oxalate absorption results from fat malabsorption [8, 12]. Under normal conditions, oxalate is rendered relatively insoluble by binding to calcium in the colon, forming a calcium oxalate complex. An increase in free fatty acids (FFAs), results in calcium binding to these FFAs instead of oxalate. As a consequence, there is an increase in soluble oxalate which is absorbed from the colon [13]. Bile salts and gut flora may also play a role in propagating this effect. Once absorbed, if the urinary oxalate concentration is such that calcium oxalate exceeds its solubility then crystals will precipitate. Although hyperoxaluria is a well-recognised cause of nephrolithiasis [14], it is also increasingly known to cause chronic kidney disease [8, 12, 15–17]. Precipitation of calcium oxalate in renal tubules and interstitium leads to direct tubular injury and atrophy, and excites chronic interstitial inflammation leading to fibrosis, and ultimately nephron loss [12]. As renal function deteriorates, oxalate clearance falls, causing higher plasma and tissue levels, often leading to inexorable decline in renal function [8]. The relationship between fat malabsorption, hyperoxaluria and chronic tubulointerstitial injury has been established in a number of case series in patients undergoing surgery to the gastrointestinal tract (e.g. ileal resection for Crohn’s disease or Roux-en-Y gastric bypass surgery for obesity) [8, 15], following pancreatic surgery [17], with pancreatic carcinoma [6] and with chronic pancreatitis [12, 16].

The case presented here also illustrates several other features of oxalate nephropathy. Firstly, similar to the classical myeloma kidney in which precipitation (this time of immunoglobulin light chains) in renal tubules and interstitium also exaggerates renal damage in response to hypovolaemia or haemodynamic compromise, patients with hyperoxaluria often display increased sensitivity to such renal insults. Secondly, recovery after such injury may be more limited than might be observed following a purely ischaemic insult of similar intensity and this patient only had partial recovery of renal function following his hypovolaemic insult. Thirdly, as eGFR falls, oxalate concentrations in the blood and kidney will rise leading to more ready precipitation. This may produce a “tipping point” beyond which progression of renal impairment can accelerate as seen in this case. Finally, while chronic interstitial fibrosis and tubular atrophy is a non-specific finding in chronic renal impairment that may be associated with minor deposition of oxalate crystals, it is the degree and extent of such precipitates that should raise the suspicion of an oxalate nephropathy.

### Pancreatic insufficiency in metastatic renal carcinoma

Although pancreatic metastases are rare, the most common primary source is a renal primary [18]. Pancreatic metastases can present years after a radical nephrectomy. Retrospective studies suggest patients with pancreatic metastatic renal carcinoma demonstrate an improved overall survival and response to targeted therapies, and tend to follow a more slowly progressive clinical path, as seen in this patient [2, 19–22]. As such, the potential for this group of patients to develop pancreatic insufficiency and sequelae such as oxalate nephropathy should be noted. In this patient group, low grade diarrhoea is often presumed to be a consequence of TKI therapy and managed using supportive medication. It is possible that earlier recognition and intervention may be able to halt or prevent decline in renal function.

### Tyrosine kinase inhibitors (TKIs) and renal failure

Tyrosine kinases are a large family of enzymes that catalyse the addition of phosphate groups to cellular proteins and have diverse roles in transmembrane signalling and signal transduction in biological processes that include angiogenesis, proliferation, survival, differentiation [23]. As such they have become important targets for the treatment of many cancer types and tyrosine kinase inhibitors (TKIs), including Pazopanib, Sunitinib, Cabozantinib and Tivozanib are currently approved as first line treatment in metastatic renal cancer [24]. Each has a differing spectrum of activity against the numerous tyrosine kinase enzymes, which likely accounts, at least in part, for the differential responses and side-effects observed.

Renal impairment is a common and important side-effect of TKIs with a variable frequency, that ranges from 30 to 70% that depends partly on the agent used; pazopanib having the lowest incidence of raised creatinine (around 30% all grades) and tivozanib the highest (70% all grades) and partly on the presence of pre-existing renal impairment. This is commonly low-grade, reversible and results from disturbances in fluid balance caused by excessive gastrointestinal losses. However, TKIs may also have more direct effects on the kidneys as manifest by the increased incidence of both hypertension and proteinuria [25–27]. These are thought to arise at least in part by disrupting glomerular VEGF signalling between podocytes and endothelial cells [25] and may lead to podocytopathies including minimal change disease or collapsing focal segmental glomerular sclerosis or to a thrombotic microangiopathy [25, 27–29]. These are important to distinguish, since they may lead to progressive and irreversible renal impairment if the tyrosine kinase inhibitor is continued.

Tyrosine kinase inhibitors are effective in patients with chronic renal impairment and although the risk of renal complications is increased in patients with pre-existing renal impairment, this does not preclude their use if due attention is paid to fluid status, blood pressure and monitoring of renal function and proteinuria [30–32]. Pazopanib and Sunitinib are largely hepatically metabolised and are not removed by haemodialysis [33], so no dose adjustment is required for renal impairment or in patients on dialysis. Although there are no large published studies or systematic reviews of the safety and efficacy of TKIs in patients with ESRF, the limited evidence available suggests that their use remains safe and effective in this patient group [34, 35] and our patient continues to tolerate pazopanib well on haemodialysis.

## Summary

This study describes a case of oxalate nephropathy in a patient treated for metastatic renal carcinoma. In the presence of a metastatic pancreatic lesion, this may explain the development of chronic pancreatic insufficiency, hyperoxaluria and oxalate nephropathy. This demonstrates the importance of considering renal biopsy in patients in whom the cause of progressive renal failure is unclear, and that treatment with TKIs can be continued safely under close observation, as in this case.

## Data Availability

Not applicable.

## Abbreviations

ATN: Acute tubular necrosis
eGFR: estimated glomerular filtration rate
FFA: Free fatty acid
IFTA: Interstitial fibrosis and tubular atrophy
OGD: Oesophago-gastroduodenoscopy
PDL-1: Programmed death ligand 1
RCC: Renal cell carcinoma
TKI: Tyrosine kinase inhibitor
uPCR: urinary protein-to-creatinine ratio
VEGF: Vascular endothelial growth factor
